# Developmental programming of polycystic ovary syndrome (PCOS): prenatal androgens establish pancreatic islet α/β cell ratio and subsequent insulin secretion

**DOI:** 10.1038/srep27408

**Published:** 2016-06-06

**Authors:** S. Ramaswamy, C. Grace, A. A. Mattei, K. Siemienowicz, W. Brownlee, J. MacCallum, A. S. McNeilly, W. C. Duncan, M. T. Rae

**Affiliations:** 1 School of Life, Sport and Social Sciences, Edinburgh Napier University, Edinburgh EH11 4BN, UK; 2 MRC Centre for Reproductive Health, The Queens Medical Research Institute, The University of Edinburgh, Edinburgh EH16 4TJ, UK

## Abstract

Exogenous androgenic steroids applied to pregnant sheep programmes a PCOS-like phenotype in female offspring. Via ultrasound guidance we applied steroids directly to ovine fetuses at d62 and d82 of gestation, and examined fetal (day 90 gestation) and postnatal (11 months old) pancreatic structure and function. Of three classes of steroid agonists applied (androgen - Testosterone propionate (TP), estrogen - Diethystilbesterol (DES) and glucocorticoid - Dexamethasone (DEX)), only androgens (TP) caused altered pancreatic development. Beta cell numbers were significantly elevated in prenatally androgenised female fetuses (P = 0.03) (to approximately the higher numbers found in male fetuses), whereas alpha cell counts were unaffected, precipitating decreased alpha:beta cell ratios in the developing fetal pancreas (P = 0.001), sustained into adolescence (P = 0.0004). In adolescence basal insulin secretion was significantly higher in female offspring from androgen-excess pregnancies (P = 0.045), and an exaggerated, hyperinsulinaemic response to glucose challenge (P = 0.0007) observed, whereas prenatal DES or DEX treatment had no effects upon insulin secretion. Postnatal insulin secretion correlated with beta cell numbers (P = 0.03). We conclude that the pancreas is a primary locus of androgenic stimulation during development, giving rise to postnatal offspring whose pancreas secreted excess insulin due to excess beta cells in the presence of a normal number of alpha cells.

Polycystic ovary syndrome (PCOS) is one of the most commonly found endocrinopathies in women during their reproductive years^1^. Whilst mechanisms underpinning the developmental route culminating in PCOS in women are unclear, animal models designed to recreate PCOS associated pathologies have utilised over-exposure of female fetuses/pregnant dams to androgenic steroids^2^,^3^,^4^,^5^,^6^,^7^,^8^,^9^,^10^. This single intervention recreates both reproductive and metabolic features of human PCOS and, given hyperandrogenic states of PCOS sufferers, may also be replicative of the human condition, evidenced by cord blood concentrations of daughters born to PCOS-patients exhibiting male ranges of androgens^11^.

PCOS has an associated raft of metabolic dysfunctions in addition to reproductive phenotypes. Sufferers are often more insulin resistant than non-PCOS patients, and as such develop compensatory hyperinsulinaemia^4^,^12^. However, both our ovine model, and a rhesus monkey model, have indicated that abnormal pancreatic function likely precedes development of insulin resistance^9^,^10^. Additionally, as opposed to simply a compensatory response to IR, recent studies place hyperinsulinaemia upstream, and contributory to, diet-induced obesity^13^. Given repeated indications of adult beta cell dysfunction in PCOS patients^14^,^15^, and the profound effects that insulin has on numerous tissue types, including promotion of ovarian steroidogenesis^16^, the possibility that the pancreas is a primary locus of initial PCOS development with a role in downstream metabolic and reproductive dysfunction warrants further investigation.

Mid-gestational androgenic over-exposure is associated with altered fetal pancreatic beta cell gene expression, predictive of increased beta cell function/proliferation, and congruent increased *in vitro* insulin secretion^9^. Increased adolescent offspring beta cell numbers in our maternally androgen exposed model was observed, and in line with this a recent series of studies in monkeys observed increased pancreatic beta cell content in infants in response to androgenic over-exposure in fetal life^10^. However, it is uncertain whether or not such beta cell proliferation originated during fetal life. Given that effects on the pancreas may reflect a contribution of both androgenic effects directly on the pancreas/beta cells, and a transient state of maternal hyperglycaemia associated with androgenic treatment of dams^10^, we developed a complimentary, direct fetal androgen exposure paradigm in order to avoid androgenic effects on pregnant dams^7^,^9^. Here we test the hypothesis that pancreatic effects of androgen over-exposure are direct consequences of androgen-driven developmental alterations, with postnatal metabolic consequences underpinned by a permanently altered endocrine pancreatic phenotype.

## Materials and Methods

### Ethics statement

All studies were approved by the UK Home Office and conducted under approved Project Licence PPL 60/4401, reviewed by The University of Edinburgh Animal Research Ethics Committee.

### Animals and fetal tissue collection

Mature Scottish Greyface ewes were fed in order to achieve a comparable body condition score (2.75–3) prior to estrous cycle synchronisation. After a synchronised mating (Texel ram) animals were allocated at random to one of two experimental groups – vehicle control or testosterone propionate (TP) exposed. On day 62 and day 82 of gestation, anaesthesia was induced by initial sedation via administration of 10 mg Xylazine i.m (‘Rompun’, Baylor plc Animal Health Division, UK), followed by 2 mg/kg ketamine (i.v, Keteset, Fort Dodge Animal Health, UK). All downstream procedures were conducted under surgical aseptic conditions. TP was dissolved in vegetable oil (100 mgml^−1^) and a 200 μl volume injected (20 G Quinke spinal needle, BD Biosciences) via ultrasound guidance into the fetal flank. Control fetuses received 200 μl vegetable oil vehicle alone. In a separate set of studies, TP was replaced with diethylsilbesterol (DES^17^) or dexamethasone (DEX 500 μgml^−1^). This procedure was then repeated at d82 of gestation. Immediately after surgical procedure completion all pregnant ewes were given prophylactic antibiotics (Streptacare, Animalcare Ltd, UK, 1 ml/25 kg) and were then monitored during recovery; no adverse effects of these procedures were observed.

In the case of fetal tissue collection, ewes were sacrificed on d90 of gestation via barbiturate overdose. The gravid uterus was immediately exteriorised, fetal sex recorded and fetal pancreatic tissue removed. Each pancreas was divided into two portions laterally; one portion was then snap frozen and stored at −80 °C, the other was immersion fixed in Bouins solution for 24 hours, rinsed free of excess Bouins with 70% ethanol, and then stored in 70% ethanol prior to processing and paraffin wax embedding for histological analysis.

In experiments where pregnancies carried to term, offspring were lambed and reared conventionally (weaned at 3 months postnatal age). Due to economic and practical constraints, only female offspring were studied. Prior to sacrifice at 11 months of age, all animals received a bolus injection of glucose (10 g glucose in 20 ml saline), and 15 minutes post injection sacrifice was achieved via barbiturate overdose. Tissues (pancreas, muscle and liver) were recovered and processed for downstream analyses as described for fetal samples.

All animal numbers are given in figure legends. Animal studies utilised twin and singleton pregnancies, but in order to avoid any possibility of genetic bias included only one animal from each pregnancy.

### Glucose tolerance testing

Animals were fasted overnight and a glucose tolerance test (GTT) performed by administering a bolus glucose injection (10 g glucose in 20 ml saline) administered intravenously following collection of a basal blood sample. Post-glucose administration, blood was sampled at 15 minute intervals for 30 minutes.

### Plasma analyte determinations

Insulin concentration in blood samples collected during GTT was determined as previously described^9^. Insulin ELISA assay sensitivity was 0.14 ng/ml and inter and intra assay CVs were <6% and <5% respectively. Glucose concentrations were determined via a colorimetric glucose assay kit (Alpha Laboratories Ltd., Eastleigh, UK), in combination with a Cobas Fara centrifugal analyzer (Roche Diagnostics Ltd., Welwyn Garden City, UK). Assay sensitivity was 0.2 mmol/l and inter and intra assay CVs were <3% and <2%, respectively.

### Examination of Gene Expression by Quantitative (Q) RT-PCR

Frozen fetal pancreas and offspring liver and striated muscle was homogenised in RLT buffer (including 2-mercaptoethanol, 1% v/v) (Qiagen, Crawley, UK) and RNA was extracted using RNeasy minispin columns following the manufacturer’s protocols, including on-column DNaseI digestion (Qiagen, Crawley, UK). RNA concentrations were determined using a NanoDrop 1000 spectrophotometer (Thermo Fisher Scientific, Loughborough, UK), with Agilent Bioanalyser analysis utilised for RNA quality control. Complimentary DNA (cDNA) was synthesised according to kit manufacturer protocols (PrimerDesign Ltd, UK). Previously published and validated primer pairs were used^7^. Quantitative PCR was performed exactly as previously described^9^, utilising Genorm analysis (PrimerDesign Ltd, UK) in order to identify a panel of three stable housekeeping genes – thus the geometric mean of *GAPDH, ATP5B* and *ACTB* was used as the normalisation reference. Ovine placental cDNA and pooled ovine fetal pancreatic cDNA were used as positive controls, and negative controls consisted of an RT-ve and a template negative reaction.

### Histology

Three sections (5 μm) were collected from each pancreatic tissue sample, a minimum of 100 μm apart, and mounted on positively charged slides (Superfrost Plus Gold, Thermoscientific, Epsom, UK). Insulin and glucagon localisation was performed as previously described^9^, using anti-insulin mouse monoclonal antibody (Abcam, Cambridge, UK; 5 μgml^−1^) or anti-glucagon rabbit polyclonal antibody (Millipore, UK; 1 in 2000 dilution). Negative controls consisted of substitution of primary antibody with mouse IgG or non-immune rabbit serum at the same concentration as the respective primary antibodies. In the case of dual fluorescent staining, anti-insulin and anti-glucagon antibodies were applied simultaneously (anti-insulin 5μgml^−1^; anti-glucagon 1 in 1000 dilution, 2% normal goat serum (NGS) as diluent). Secondary detection reagents consisted of biotinylated goat anti-rabbit (Vector Laboratories, UK, 1 in 500 dilution in 2% NGS) in combination with Dylight 549 streptavidin (Vector Laboratories, UK, 1 in 100 dilution in 2% NGS) and Alexafluor 488 goat-anti-mouse IgG (Life Technologies, UK, 1 in 100 dilution in NGS), followed by washing procedures^9^ and final mounting in aqueous mountant containing DAPI (Vectashield, vector laboratories, UK), prior to visualisation on a Zeiss LSM 880 AxioObserver Z1 confocal fluorescent microscope (wavelengths: 405 nm, 488 nm, 594 nm, laser power set at 2%).

### Cell quantification

Each section generated by standard chromagen immunohistochemistry was subjected to quantification by an operator blinded to treatment. Five fields on each section were selected at random, and numbers of stained cells within an eye piece grid counted, and a mean count per mm^2^ derived for each animal.

### Western blotting

Western blot analysis of liver and muscle, collected 15 minutes post bolus glucose administration at sacrifice was carried out as previously described^9^. After confirmation of molecular weight by molecular weight markers, blots were scanned with an Odyssey^®^ Infrared Imaging System (LI-COR Biosciences, Cambridge, UK) using appropriate filters (700/800 nm channel) and proteins bands numerically quantified using Image Studio Lite™ v.2.0 software package.

### Statistical analyses

All analyses were conducted using GraphPad Prism v6.0 (GraphPad Software Inc., San Diego, CA), regarding values of *P* < 0.05 as significant (alpha = 0.05). Animal numbers are given in figure legends, and were based upon previous observations of maternal androgenisation paradigms generating significant, meaningful differences from n = 4–6 per treatment group^7^,^8^,^9^. Data were logarithmically transformed prior to analysis where non-Gaussian distributions were indicated or unequal variabilities indicated by Brown-Forsythe testing. Unpaired, two-tailed student t-test was used throughout where only single comparisons were made, multiple comparisons were performed by one way ANOVA with Tukeys post-hoc testing utilised for multiple comparisons. Area under the curve (AUC) was calculated using the trapezoidal method. Correlation was assessed by calculation of the Pearson r co-efficient. Insulogenic index was calculated by published method^18^.

## Results

### Direct exposure to testosterone prenatally and insulin secretion in adolescent females

**Figure 1:**
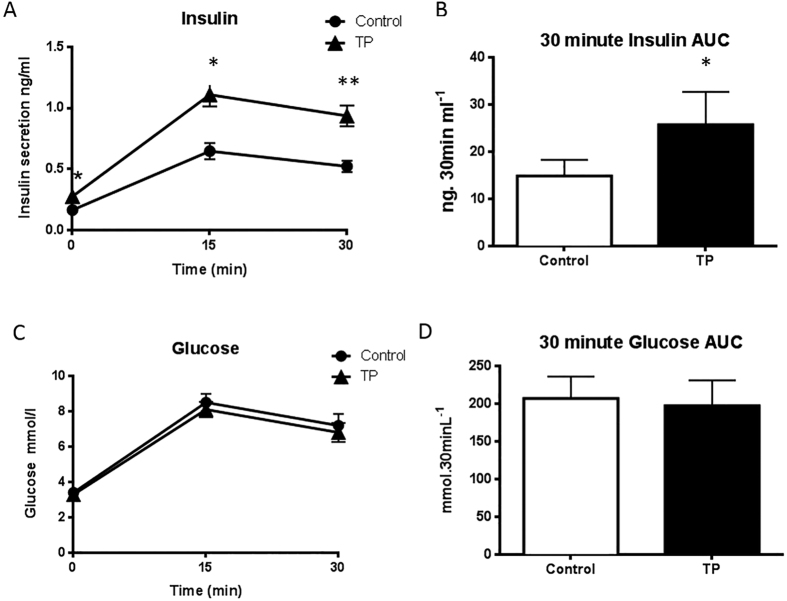
Prenatal androgen excess alters insulin secretion in adolescent offspring. Intravenous GTT was performed on 11 month old female offspring from control (n = 6) and fetal TP exposed (n = 13) pregnancies. Basal insulin was elevated slightly in prenatal TP ewes, and an exaggerated response in terms of insulin was noted at 15 and 30 minutes post-glucose injection in TP-exposed animals as compared to controls (**A**), leading to significantly increased insulin secretion over the duration of the test as calculated by AUC (**B**). There were no alterations in glucose concentrations at any time point measured after glucose administration (**C**), and no differences in glucose concentration over the duration of the test as measured by AUC (**D**). (*P < 0.05; **P < 0.01). Data represents mean ± s.e.m.

Direct injection of androgen into the developing female fetus had no impact on weight at birth (C: 5.8 ± 0.32 kg; TP: 5.9 ± 0.27 kg) or adolescence (C: 42.4 ± 3.37 kg; TP: 45.57 ± 3.94 kg). In adolescence basal (fasting) insulin concentrations were mildly elevated (*P* = 0.045) by prenatal androgen treatment, and there was a markedly exaggerated insulin response at both 15 (*P* = 0.0017) and 30 minutes (*P* = 0.0009) post-glucose administration in ewes which were injected with TP during fetal life as compared to vehicle treated control animals (Fig. 1A). Over the duration of the 30 minute GTT there was no difference in AUC_glucose_ (Fig. 1C,D), however AUC_insulin_ was significantly elevated in prenatal TP treated ewes as compared to vehicle controls (*P* = 0.0007) (Fig. 1B). In female offspring hyperinsulinaemia can therefore be directly prenatally programmed by testosterone.

### Direct exposure to testosterone prenatally and insulin action in adolescent females

We next examined if offspring hyperinsulinaemia was associated with alterations in peripheral insulin reception at a molecular level. Western blotting for total and phosphorylated ERK and AKT/PKB was performed on liver and muscle biopsies collected 15 minutes post-glucose bolus administration. There were no significant differences associated with prenatal treatment in ERK or AKT phosphorylation in either muscle or liver (Table 1). In addition we did not observe any significant differences in gene expression of key insulin response genes/glucose transporters in either tissue (Table 1). There is an apparent lack of molecular motifs associated with insulin resistance (IR) in the hyperinsulinaemic adolescent female offspring directly exposed to testosterone *in utero*.

### The relationship between hyperinsulinaemia and pancreatic islet morphology in adolescent females

**Figure 2:**
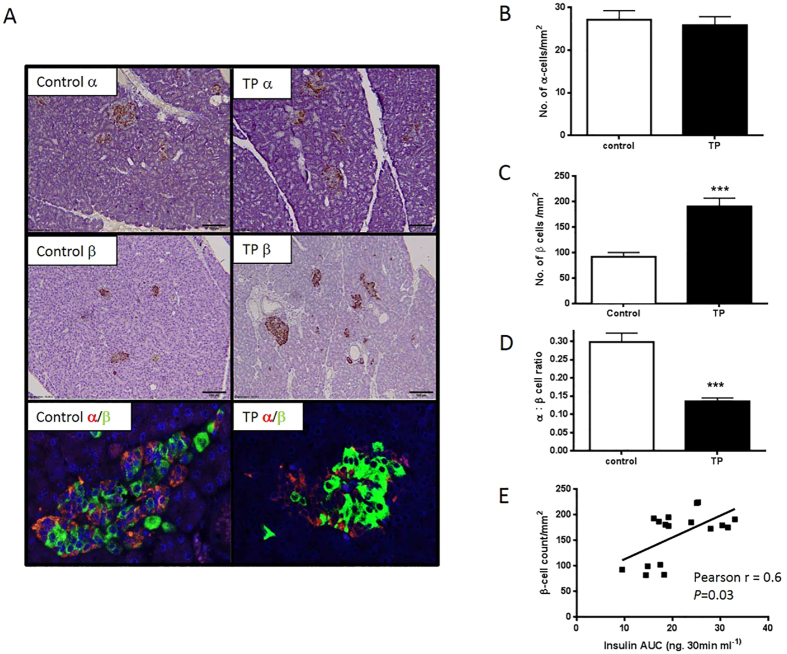
Prenatal androgen excess and altered pancreatic structure in postnatal offspring underpins increased insulin secretion. Representative immunohistochemistry images from 11 month old control and direct TP treated female offspring (panel A). Top panels show pancreatic tissue stained for glucagon indicating alpha cells. The middle two panels show beta cells after immunostaining for insulin. The lower two panels show immunofluorescence of alpha cells (red) and beta cells (green) demonstrating beta cell dominance in islets from *in utero* TP treated offspring as compared to control offspring pancreas tissue at 11 months postnatal age. Scale bars = 50 μm. No effects of prenatal treatment occurred as regards alpha cells (Panel B), but beta cell counts were significantly elevated as compared to control animals (Panel C). This depressed the alpha:beta cell ratio in TP treated female fetuses (**D**). There was a significant correlation (P = 0.03) between the amounts of insulin secreted over a thirty minute GTT (see Fig. 1) and the number of beta cells quantified (**E**). (***P < 0.001). Data represents mean ± s.e.m.

Alpha and beta cells in pancreatic islets were identified by immunohistochemistry and quantified. Direct fetal injection of TP had no effect on the islet alpha cell content in adolescent females (Fig. 2A,B). However there was a marked elevation in the numbers of beta cells in the pancreatic islets of female offspring treated with TP prenatally as compared to offspring injected with vehicle control *in utero (P* = 0.009 × 10^−9^) (Fig. 2C). Consequently direct exposure to excess testosterone prenatally was associated with a decreased alpha:beta cell ratio in the pancreatic islets of female offspring in adolescence (*P* = 0.0004) (Fig. 2D). There was a direct relationship between beta cell numbers and insulin secretion over a 30 minute GTT (Pearson *r* = 0.6; *P* = 0.03, Fig. 2E). Furthermore, the insulogenic index was elevated in adolescent females who were prenatally injected with TP as compared to vehicle-treated controls animals (C: 0.49 ± 0.12; TP 0.76 ± 0.07 (*P* = 0.034). The increased insulin response to glucose is associated with alterations in pancreatic islet morphology.

### Sex differences in fetal pancreatic islet development

**Figure 3:**
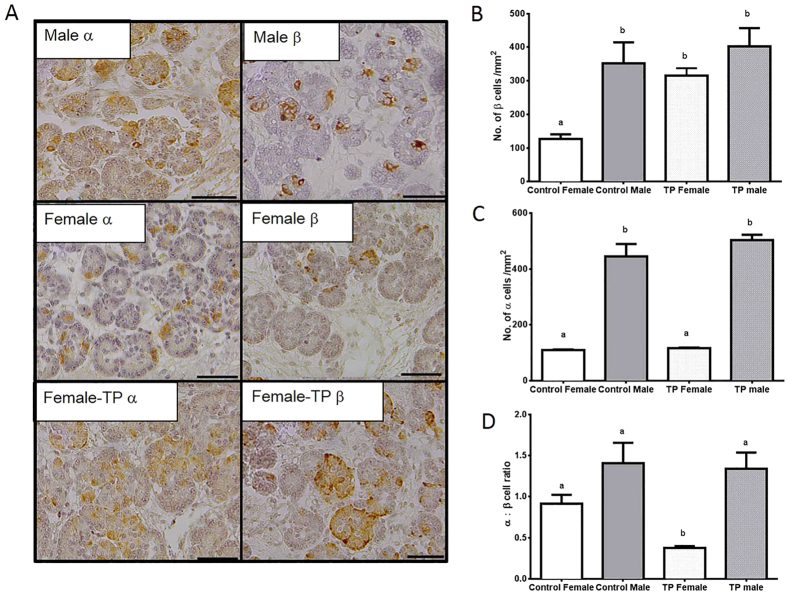
Sex differences in fetal pancreatic development are partially recreated by prenatal androgen excess in females. Immunohistochemistry images highlighting staining (brown) for insulin (beta cells) and glucagon (alpha cells) in female and male fetal pancreatic islets (Panel A). Quantification of immunostained pancreatic beta (**B**) and alpha (**C**) cells respectively, in d90 gestation fetuses from control (FC: n = 5, MC n = 5) and TP treated (FTP: n = 5, MTP n = 5) female and male fetuses. Male fetuses had significantly higher numbers of both alpha and beta cells than female fetuses, however TP treated females had a similar beta cell profile to males. This in turn depressed the alpha:beta cell ratio in TP treated female fetuses (**D**). Differing superscripts indicate significant differences (*P* < 0.01−*P* < 0.001, see text for details). Data represents mean ± s.e.m.

We next hypothesised that there were differences in male and female pancreatic development, mediated by androgens, and that prenatal androgens programmed a male islet phenotype, which was discordant with the postnatal environment. We therefore quantified alpha and beta cells numbers in fetal pancreatic islets (Fig. 3A). At day 90 gestation male fetuses have increased beta cell numbers when compared to female fetuses (Fig. 3B; ANOVA *P* = 0.0021, control female vs control male *P* = 0.01). Injection of testosterone into female fetuses resulted in an increase in beta cells (*P* < 0.05) to male-like concentrations suggesting that this increase is androgen dependent (Fig. 3B). Indeed AR expression in the fetal pancreas was higher in males than females (MC: 0.89 ± 0.22; FC: 0.35 ± 0.03; *P* = 0.012) and, as a marker of ‘male’, was increased in females in response to testosterone exposure (FTP 0.51 ± 0.06; *P* = 0.034). There was no further increase in beta cells when male fetuses were injected with testosterone (Fig. 3B).

Male fetuses also had a significantly higher number of alpha cells as compared to female fetuses (ANOVA *P* = 0.013, control male vs control female *P* < 0.001) (Fig. 3C). Thus there were no sex differences in the islet alpha:beta cell ratio, which in the day 90 fetal pancreas is approximately 1:1 (Fig. 3D). Direct injection of testosterone into female fetuses had no effects upon alpha cell numbers (Fig. 3C). Thus in fetal life direct exposure to testosterone alters the female fetal alpha:beta cell ratio in fetal islets as compared to control females (*P* < 0.01) and control males (*P* < 0.01) (Fig. 3D ANOVA *P* = 0.0017). This alteration is reminiscent of that observed in the adolescent offspring (Fig. 2D).

### The effect of other steroid classes on pancreatic islet development and function

**Figure 4:**
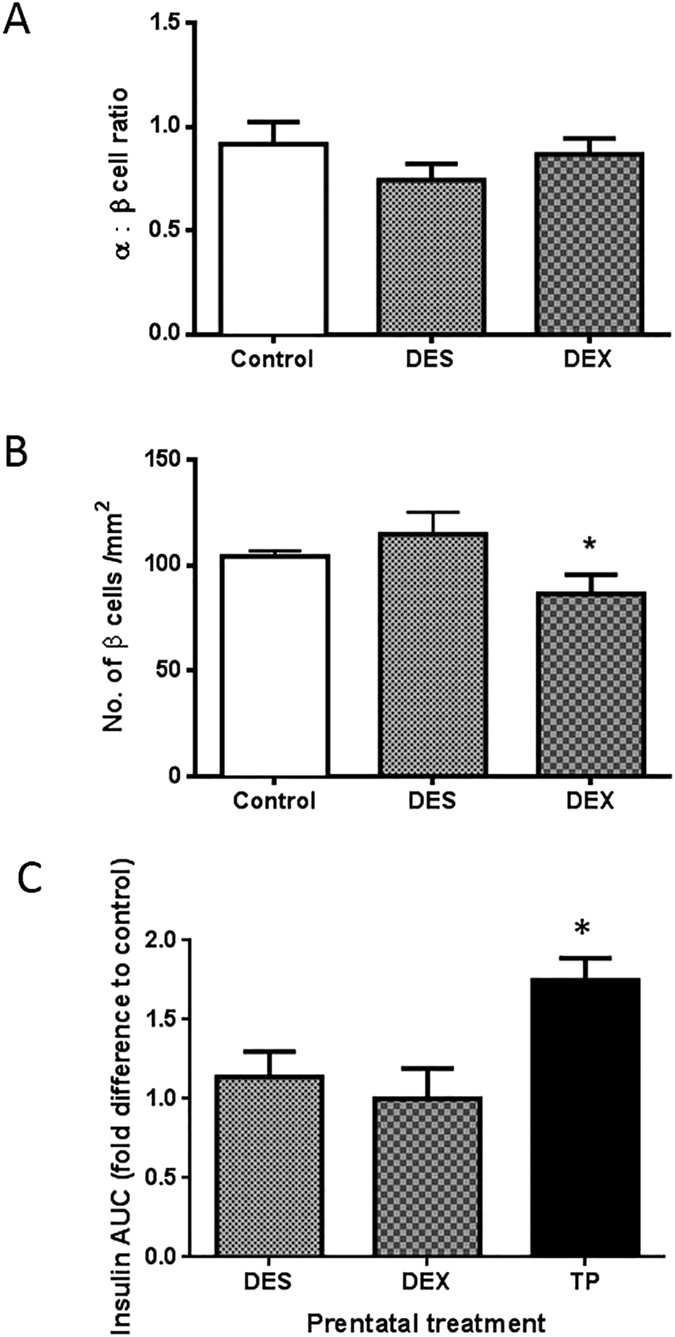
Altered pancreatic structure and function in female offspring is specifically associated with prenatal androgen excess only. When experiments were repeated substituting DEX (n = 4) or DES (n = 4) in place of TP, there was no alteration in fetal alpha:beta cell ratio associated with either treatment when compared to control (vehicle treated) animals (n = 5) (Panel A). In adolescence, *in utero* DES (n = 4) treatment was not associated with altered beta cell numbers, however, there was a small decrease in beta cell numbers associated with DEX (n = 9) treatment in utero as compared to (vehicle) control animals (n = 6) (**B**). Panel C shows the fold change against vehicle controls in AUC insulin secretion over a 30 minute GTT. Of the three steroid classes examined, only prenatal androgens elicited a significant change in insulin secretion. (*P < 0.05). Data represents mean ± s.e.m.

We further hypothesised that there may be indirect effects of testosterone in the female fetus, either by affecting adrenal function or through local metabolism to estrogen, that may be involved in programming islet development. Therefore to fully confirm that the effect on the islet development is specific to testosterone we assessed the effect of fetal injection of estradiol and cortisol agonists. Direct fetal injection with DES or DEX showed no alteration in the fetal pancreatic islet alpha:beta cell ratio, unlike the altered profile caused by direct injection of TP (Fig. 4A). In addition there was no alteration in the adolescent female islet β-cell numbers associated with DES treatment, however there was a small, but nonetheless statistically significant reduction in adolescent beta cell numbers associated with prenatal DEX treatment (*P* = 0.01) (Fig. 4B). Only prenatal TP was associated with altered insulin secretion in adult life (*P* < 0.05 as compared to DES and DEX) (Fig. 4C ANOVA *P* = 0.0057).

## Discussion

PCOS in women is associated with post-pubertal IR development^19^, thereby promoting compensatory hyperinsulinaemia^4^,^12^. In fetal androgenic over-exposure ovine models, designed to phenocopy diagnostic criteria for PCOS, female adolescent offspring hyperinsulinaemic responses to glucose administration are observed in the absence of measurable IR^7^,^9^. Similarly, excessive insulin secretion (when measured relative to insulin sensitivity) precedes altered insulin sensitivity in (prenatal androgenic over-exposure) monkey models of PCOS^5^. These observations in animal models of PCOS identify the Islets of Langerhans as a potential primary loci of downstream metabolic perturbations caused by fetal androgenic over-exposure. Given mechanistic relationships between insulin excess and ovarian androgen excess^16^,^20^, it is critical to understand the aetiological relationship between these metabolic and reproductive interfaces in PCOS.

Partial alignment of fetal female pancreatic beta cell-associated gene expression with that seen in fetal males in response to androgen-over-exposure commencing in the second trimester has been observed^9^. Masculinisation of hypothalamic function in terms of regulation of energy intake has been observed, and ascribed to prenatal steroid differences between males and females during development^21^. The male beta cell profile noted was likely created by endogenous, intra-fetal androgens derived from the developing testes, supported herein by the masculinisation of pancreatic AR expression in response to androgen excess in the developing female fetus. This, and other previously reported gene expression shifts, is predictive of fetal islet development being masculinised by prenatal androgen concentrations. However, whilst previous experiments reported structural alterations in postnatal ovine pancreases associated with increased maternal testosterone concentrations, fetal antecedents of this phenomenon remained equivocal, regardless of fetal gene expression and *in vitro* insulin secretion being predictive of postnatal alterations observed^9^. Maternal injection of testosterone caused both testosterone and estradiol elevation^9^, and lack of fetal dosage control in maternally applied steroids enhances variability in outcome, hence we have re-examined the fetal pancreas in a controlled, direct fetal steroid injection model.

Fetal males had significantly greater numbers of both alpha and beta cells than females. Alpha cell counts were unaltered by over-exposure to TP during female fetal development, and thus if testicular androgens configured alpha cell proliferation in males then sensitivity to androgens is diminished by mid gestation. Conversely, beta cell numbers, higher in males than in females, were elevated in exposed female fetuses to a similar profile to that of males, hence our androgen treatment regimen specifically masculinised the beta cell profile alone. We have previously demonstrated ovine fetal beta cells to express androgen receptors^9^, and since this was direct fetal administration of TP it would appear that this is a direct androgenic effect; in this regard testosterone can act in an anti-apoptotic protective manner towards beta cells^22^, in addition to predicted pro-proliferative effects from gene expression analyses^9^.

There was no effect in fetal males in terms of altered islet cell numbers in response to TP over-exposure, presumably since the male has a ‘buffering capacity’ against exogenous androgens, via suppression of endogenous T production from the testes^17^. Importantly, regardless of absolute cell number differences between sexes, both control male and control female ovine fetal pancreases display an approximately 1:1 alpha:beta cell ratio, which is markedly reduced by direct androgenic over-exposure in female fetuses. This 1:1 alpha:beta cell ratio is also apparent in female rhesus monkey infants, and their alpha:beta cell ratio is perturbed in a similar fashion to that observed here, in response to prenatal, maternal androgen excess^10^.

Critically, both routes of androgen administration, indirect maternal and direct fetal, culminate in similar pancreatic postnatal outcomes, but here, using the reductive approach of direct fetal exposure, we reveal alterations in fetal life that underpin the observed postnatal outcomes, attributable to excess androgen. Since maternal androgen excess models utilising rhesus monkeys^10^ and sheep^9^, both also indicated altered islet size, then this combined with the similarly perturbed alpha:beta cell ratios in both sheep and monkey models during early postnatal life further validates ovine models in terms of the ‘gold standard’ non-human primate model, and thus human translation, however, we acknowledge that *in utero* steroid environments may differ to some extent between sheep and humans, principally due to differences in the functional steroidogenic maturation and pituitary drive of the adrenal cortex between these two species^23^,^24^.

In order to comprehend longevity and health-relevance of androgen-excess fetal life perturbations, we analysed alpha and beta cell populations in postnatal (11 month) adolescent offspring. The androgenised fetal beta cell pancreatic phenotype persisted throughout early postnatal life and beyond puberty, with similar reductions in alpha:beta cell ratios. In both fetal and postnatal life, effects upon pancreatic structure/beta cell numbers were specifically a response to excess androgens. Whilst maternal androgenisation culminates in increased fetal androgen and estrogen concentrations we observed no effects of DES on fetal alpha:beta cell ratio or postnatal beta cell numbers. Prenatal DEX treatment was associated with a modest reduction in postnatal beta cell numbers. The potential significance of this remains unknown, however, the importance herein is that since DEX is associated with decreased beta cells, the aforementioned androgenic effects are direct, not associated with either metabolism of androgens or altered fetal adrenal function.

We were unable to detect development of IR at a molecular level in the liver or striated muscle. Altered insulin signalling in PCOS, (Ras-ERK phosphorylation) has been reported and linked to IR/hyperinsulinaemia^25^. However we did not observe any significant alterations in genes encoding glucose transporters or insulin signalling molecules in muscle or liver, or indeed insulin signalling alterations in terms of phosphorylation of insulin receptor MAPK or AKT/PKB signalling pathways, in line with our previous observations^9^. Nicol *et al*. noted that in later postnatal life altered cell ratio’s observed in prenatally androgenised rhesus monkey infants were no longer present^10^. Given that these monkeys are by this stage of their lives insulin resistant, and our sheep are not, we hypothesise that the altered beta cell profile we observe in early adulthood reflects a pancreas altered in fetal life, but as yet not placed under any metabolic pressure from developing IR.

PCOS patients display greater compensatory insulin responses to IR, leading to the suggestion that beta cell hypersecretion may be a primary PCOS characteristic that then interacts with developing IR^14^. IR-corrective weight reduction does not correct insulin hypersecretion to the same degree^26^, implying that hypersecretion of insulin may be an initial alteration in PCOS in addition to, but independent of, IR-induced beta cell compensation, persisting even when IR has been largely ameliorated. To ascertain whether or not the histological observations noted above in fetal and postnatal life have functional causatum, an intravenous GTT analysis was conducted on adolescent offspring. This revealed an exaggerated insulin response in the animals who were directly prenatally androgenised, whereas increased estrogenic or glucocorticoid exposure *in utero* had no effects upon postnatal insulin concentrations. Basal insulin, and glucose-stimulated AUC_insulin_ was significantly increased in TP treated animals, with no attendant alteration in glucose profiles. We suggest that increased beta cell content underpins this exaggerated insulin response. In support, insulogenic index was increased in adolescent offspring animals from prenatal androgenisation regimens, and we note a correlation between beta cell numbers and AUC_insulin_. Insulogenic index is also raised in women with PCOS as compared to non-PCOS patients, irrespective of obesity status^27^, and early insulin response to glucose elevated in PCOS, and resolvable from IR^28^, although it is acknowledged that there are conflicting reports of insulin secretion in PCOS^18^.

We previously demonstrated that the fetal pancreas from androgen overexposed (maternal and/or fetal administration) animals secreted excessive insulin to a fixed glucose challenge *in vitro*^9^, thus this functional characteristic is established *in utero*, and, via permanent pancreatic structural change, persists on into postnatal life. In terms of the aetiology of altered insulin/glucose dynamics, reductions in insulin sensitivity do not manifest in daughters of PCOS patients until after puberty, where although fasting insulin was not raised, 2 h-post glucose challenge insulin secretion is higher in girls whose mothers have PCOS^19^, indicative that altered insulin secretory response may also precede onset of IR in humans.

Since hyperinsulinaemia likely precedes IR development in girls born to PCOS mothers^19^, and antecedes IR in both ovine and monkey^10^ *in utero* androgen exposure models, it begs the question of whether hyperinsulinaemia could be a causative factor in the sequelae of events culminating in IR development. Excess, sustained insulin concentrations may predispose to development of IR^29^. Intriguingly, a series of studies utilising ovine maternal-androgen exposure regimens report increased insulin sensitivity at 16 months of age in T-exposed female offspring^30^, which converted to an IR state by 22 months of age^31^. We hypothesise that androgen-programmed (primary) hyperinsulinaemia may therefore act as a contribution in promotion of initial IR development. Further study is required to resolve the relative contributions of primary hyperinsulinaemia, IR development and subsequent compensatory hyperinsulinaemia in development of glucose intolerance, particularly in light of current evidence implicating hyperinsulinaemia causally to the development of obesity^13^.

In conclusion, these experiments demonstrate that androgen overexposure has a direct and permanent effect in terms of increased beta cell numbers in female pancreases. The outcome of this partial masculinisation of islets is altered pancreatic function, delivering a primary hyperinsulinaemic response to glucose. We identify the pancreas as a primary loci of downstream effects of fetal androgen overexposure, and demonstrate that the prenatal steroid environment is an important driver of future metabolic health.

## Additional Information

**How to cite this article**: Ramaswamy, S. *et al*. Developmental programming of polycystic ovary syndrome (PCOS): prenatal androgens establish pancreatic islet α/β cell ratio and subsequent insulin secretion. *Sci. Rep.* **6**, 27408; doi: 10.1038/srep27408 (2016).

## Figures and Tables

**Table 1:** Insulin signalling-associated gene expression and pathway activation are not altered in adolescent female offspring exposed to excess androgen during fetal life.

End point	Female control	Female TP	Significance
Western blot			
Hepatic *t*ERK: *p*ERK	6.88 ± 2.50	8.87 ± 3.74	NS
SM *t*ERK: *p*ERK	1.01 ± 0.49	1.47 ± 0.87	NS
Hepatic *t*AKT: *p*AKT	0.58 ± 0.16	0.75 ± 0.33	NS
SM *t*AKT: *p*AKT	0.52 ± 0.32	0.66 ± 0.07	NS
qRT-PCR			
Hepatic *SLC2A1*	2.93 ± 1.02	4.99 ± 1.44	NS
SM *SLC2A1*	0.97 ± 0.29	0.82 ± 0.12	NS
Hepatic *SLC2A4*	2.61 ± 0.62	3.98 ± 0.92	NS
SM *SLC2A4*	0.93 ± 0.2	1.18 ± 0.16	NS
Hepatic *IRS1*	4.1 ± 1.43	5.87 ± 1.73	NS
SM *IRS1*	0.40 ± 0.19	2.28 ± 0.69	NS
Hepatic *INSR*	3.09 ± 0.99	4.58 ± 1.47	NS
SM *INSR*	1.02 ± 0.17	0.61 ± 0.09	NS

Insulin signalling genes and phosphorylation of ERK1/2 and AKT were quantified in a random subset of 11 month old female offspring from control (n = 5) and TP treated pregnancies (n = 5), in skeletal muscle (SM) and hepatic tissue samples obtained at sacrifice, 15 minutes post intravenous glucose bolus administration. There was no significant (NS) effect of TP treatment upon any of the end-points quantified. Data represents mean ± s.e.m.
